# African swine fever virus protein p17 promotes mitophagy by facilitating the interaction of SQSTM1 with TOMM70

**DOI:** 10.1080/21505594.2023.2232707

**Published:** 2023-07-13

**Authors:** Boli Hu, Guifang Zhong, Shuxiang Ding, Kang Xu, Xiran Peng, Weiren Dong, Jiyong Zhou

**Affiliations:** a MOA Key Laboratory of Animal Virology, Zhejiang University Center for Veterinary Sciences, Hangzhou, PR, China; b State Key Laboratory for Diagnosis and Treatment of Infectious Diseases, Zhejiang University First Affiliated Hospital, Hangzhou, PR, China

**Keywords:** African swine fever virus, p17, mitophagy, TOMM70, SQSTM1

## Abstract

Viruses have developed different strategies to hijack mitophagy to facilitate their replication. However, whether and how African swine fever virus (ASFV) regulates mitophagy are largely unknown. Here, we found that the ASFV-encoded p17 induced mitophagy. Coimmunoprecipitation/mass spectrometry assays identified translocase of outer mitochondrial membrane 70 (TOMM70) as the protein that interacted with p17. The binding of TOMM70 to p17 promoted the binding of the mitophagy receptor SQSTM1 to TOMM70, led to engulfment of mitochondria by autophagosomes, and consequently decreased the number of mitochondria. Consistently, the levels of TOMM70 and TOMM20 decreased substantially after p17 expression or ASFV infection. Furthermore, p17-mediated mitophagy resulted in the degradation of mitochondrial antiviral signalling proteins and inhibited the production of IFN-α, IL-6 and TNFα. Overall, our findings suggest that ASFV p17 regulates innate immunity by inducing mitophagy via the interaction of SQSTM1 with TOMM70.

## Introduction

African swine fever virus (ASFV) causes African swine fever (ASF) in swine; ASFV is contagious, and its infection is associated with high morbidity and mortality rates [1]. To date, there is no effective vaccine or drug available, resulting in the rapid spread of ASFV in more than 60 countries and causing a substantial economic burden worldwide [2]. ASFV is an enveloped icosahedral virus with a double-stranded DNA genome that ranges from 170 to 193 kb in length and encodes more than 150 proteins. Most virus proteins lack any known or predictable functional information, preventing the development of clinically effective drugs and vaccines against this virus [3].

The cGAS/STING signalling pathway is critical for sensing and detecting intracellular microbial and host-derived DNA to activate innate immunity [4]. As expected, the DNA virus ASFV stimulates this signalling pathway [5,6]. However, a number of ASFV virulence factors, such as DP96R, MGF-360-11 L, MGF-505-7 R, MGF-505-11 R and pS273R, mediate strategies that block activation of the cGAS/STING signalling pathway [5,7,8]. Interestingly, the RNA sensor RIG-I-mediated signalling pathway is also activated by transcripts of AT-rich regions contained in the ASFV genome through DNA-dependent RNA polymerase III. However, this pathway is ultimately inhibited by ASFV protein I267L [9]. Activation of TLR3, a sensor of dsRNA, is also inhibited by two ASFV-encoded proteins, A276R and I329L [10,11]. Mitochondria play key roles in the innate immune response via RLR signalling and mitochondrial DAMP (damage-associated molecular pattern; such as mtDNA and N-formylated peptide)-regulated signalling [12]. Recently, a report showed that p17, encoded by the ASFV *D117L* gene, inhibits cGAS-STING signalling by binding to STING [13]. However, whether and how ASFV mediates the mitochondria-mediated innate immune response is unclear.

Autophagy is one of the main pathways for the selective degradation of damaged or excessive mitochondria. This process is termed mitophagy. Mitochondrial outer membrane (MOM) proteins, including NIX, BNIP3, FUNDC1, BCL2L13, and FKBP8, serve as receptors for targeting mitochondria to autophagosomes for eventual autophagic degradation [14]. Moreover, Parkin induces mitophagy by regulating the ubiquitination-mediated degradation of several mitochondrial proteins, such as mitochondrial fusion proteins Mfn1 and Mfn2, the mitochondrial input receptor subunits TOMM40, TOMM20 and TOMM70, hexokinase II, members of the Bcl-2 family, mitochondrial Rho GTPases and VDAC1 [15–20]. Recently, mitochondrial import factors, including the Mim1–Mim2 complex and TOMM70, were reported to be crucial for mitophagy [21]. Mitophagy plays critical roles in neurodegenerative diseases, ischaemia- or drug-induced tissue injury, and terminal differentiation of red blood cells [22]. In particular, virus-triggered mitophagy benefits viral replication by inhibiting apoptosis, type I interferon responses and inflammation [23]. However, whether a member of the TOMM core complex is an adaptor for mitophagy is still unknown.

p17 is encoded by the D117L gene and expressed late in the viral infection cycle. This protein majorly localizes at intracellular and extracellular mature virus particles, and is an integral membrane with a Singer type I topology [24,25]. p17 is highly abundant and essential for the assembly and maturation of the icosahedral capsid as well as general virus viability [26]. Together with p72, H240R, M1249L, and p49, p17 forms the outer capsid shell [27,28]. The p17 protein appears to form trimers and is located at the interface of the centre gap region of three neighbouring pseudo-hexameric capsomers [27]. P17 closely associates with the base domain of p72, and three copies of p17 encircle each p72 trimer capsomer in the inner capsid shell, firmly anchoring p72 capsomers on the inner membrane [29].

In this study, we found that p17 promoted mitophagy by facilitating the interaction between TOMM70 and SQSTM1 and inhibited the innate immune response by degrading MAVS, suggest a role for p17-mediated mitophagy in immune escape by ASFV.

## Results

### AFSV p17 promotes autophagy

**Figure 1.**
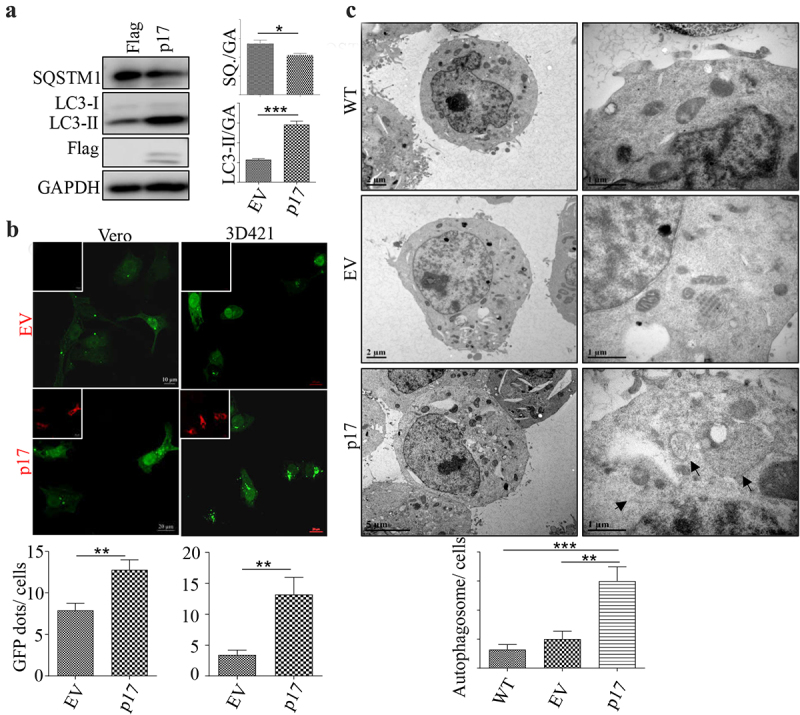
AFSV p17 promotes autophagy.

To determine whether ASFV p17 regulates autophagy, HEK-293T cells were transfected with a vector expressing p17 for 36 h, and the expression of the autophagy markers LC3 and SQSTM1 was measured by Western blotting (WB) assay. As shown in Figure 1a, the conversion of LC3-I to LC3-II increased, and the level of SQSTM1 decreased greatly, indicating that p17 induced autophagy. We then tested whether p17 induced the formation of autophagosomes by confocal analysis of GFP-LC3 puncta formation. As shown in Figure 1b, p17 expression increased the number of GFP-LC3 puncta in Vero cells and in 3D4/21 cells. We further measured autophagic activity by quantifying autophagosome number in wild-type cells or cells transfected with an empty vector or a vector expressing p17 by transmission electron microscopy (TEM). Autophagosomes are identified by the presence of two distinct membrane bilayers that are separated by an electron-translucent aperture. These structures typically measure between 0.5–1.5 μm in size and contain materials intended for degradation [30]. As shown in Figure 1c, few autophagosomes were observed in WT cells or cells harbouring empty vector, while the number of bilayer membrane structures in cells expressing p17 increased significantly (Figure 1c). The above data suggest that p17 induced autophagy.

(a) HEK-293T cells were transfected with empty vectors or plasmids expressing Flag-tagged ASFV p17. At 36 h post-transcription, cells were harvested for WB analysis by using anti-GAPDH, anti-LC3, anti-Flag, and anti-SQSTM1 antibodies. (b) Vero and 3D4/21 cells were transfected with vectors expressing ASFV p17 or empty vectors (EV). Cells were harvested at the indicated time points (48 h). IFA and confocal analysis were then performed. GFP-dots and cell numbers were counted by using ImageJ software, *n*>15. c HEK-293T cells were transfected with plasmids expressing ASFV p17 or empty vectors. At 36 h post-transcription, cells were harvested for TEM (transmission electron microscopy) analysis. The number of autophagosomes per cell was counted by a counter, *n*>15. Black arrows indicate autophagosomes. At least 3 independent experiments were performed. The error bars show the standard error of the mean (SEM). Significance was analyzed with two-tailed Student’s test. *p<0.05, **p<0.01, ***p<0.001.

### P17 interacts with the host protein TOMM70

**Figure 2.**
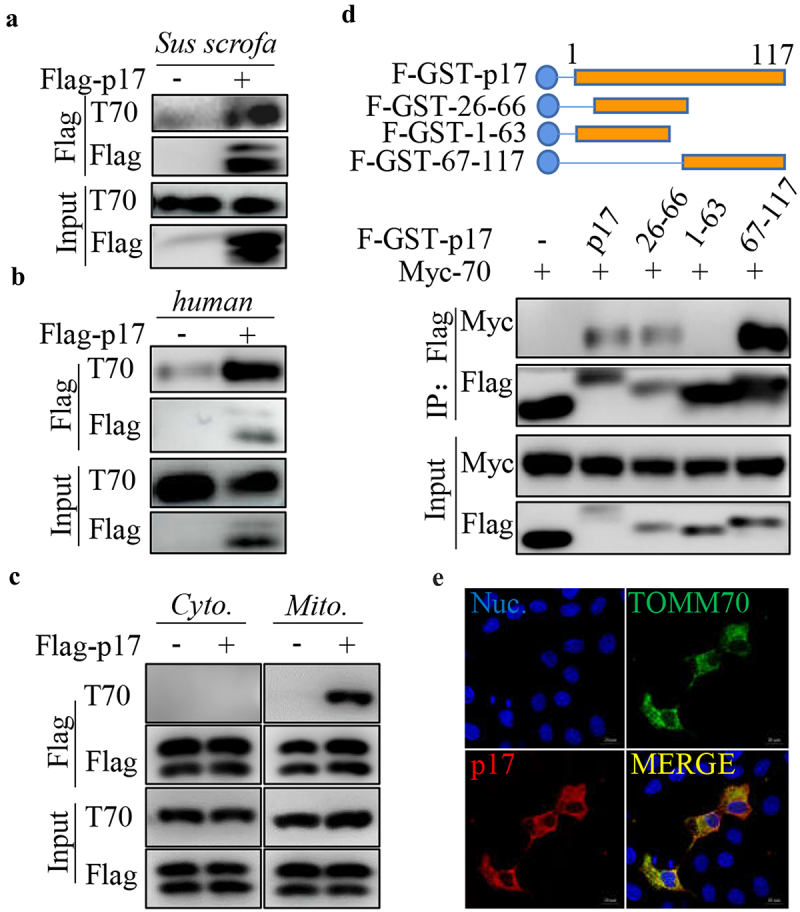
P17 interacts with TOMM70.

Our IP-MS data showed that p17 potentially interacted with TOMM70 [31]. Here, we used a coimmunoprecipitation (CO-IP) assay to evaluate the interaction between p17 and TOMM70. 3D4/21 cells or HEK-293T cells were transfected with vectors expressing Flag-p17. Anti-Flag IP was then performed. As shown in Figures 2a,b, p17 precipitated together with endogenous TOMM70 from human or *Sus scrofa* samples. To examine whether p17 specifically interacts with mitochondrial TOMM70, mitochondrial and cytoplasmic fractions extracted from HEK-293T cells transfected with an empty vector or a vector expressing Flag-p17 were subjected to anti-Flag IP. As shown in Figure 2c, p17 interacted with TOMM70 from mitochondria rather than from the cytoplasm. Moreover, to map the segments of p17 that interact with TOMM70, HEK-293T cells were cotransfected with vectors expressing Flag-GST-tagged truncated p17 and Myc-TOMM70, and GST pull down was performed. WB analysis showed that segments 26 to 66 and 67 to 117, but not segments 1 to 63, interacted with Myc-TOMM70, suggesting that theC-terminus of p17 is required for binding to TOMM70 (Figure 2d). We also tested the colocalization between p17 and TOMM70 by confocal analysis. As shown in a showed that p17 potentially inter 2E, p17 overlapped with TOMM70, confirming the interaction between p17 and TOMM70.

3D4/21 (a) or HEK-293T (b) cells were transfected with empty vectors or plasmids expressing Flag-tagged p17. At 36 h post-transcription, cells were harvested for CO-IP. WB analysis was performed by blotting with anti-TOMM70 (T70) and anti-Flag antibodies. (c) HEK-293T cells were transfected with empty vectors or plasmids expressing Flag-tagged p17. At 36 h post-transcription, cells were harvested and used for mitochondrial extraction, and the mitochondrial fraction and cytoplasmic fraction were used for anti-Flag IP. WB analysis was performed by blotting with anti-TOMM70 (T70) and anti-Flag antibodies. (d) HEK-293T cells were cotransfected with Myc-TOMOM70 (Myc-70) and each truncated Flag-GST (F-GST)-tagged P17, such as F-GST-p17 (p17), F-GST-26-66 (26-66), F-GST-1-63 (1-63), and F-GST-67-117 (67-117). GST beads were then used to precipitate each truncated mutant. WB analysis was then performed by blotting with anti-Myc or anti-Flag antibodies. (e) 3D4/21 cells were cotransfected with Flag-p17 or Myc-TOMM70. The fluorescence signals were visualized by confocal immunofluorescence microscopy, scale bars: 5 µm. At least three independent experiments were performed. The error bars show the standard error of the mean (SEM). Significance was analysed with two-tailed Student’s test. *p<0.05, **p<0.01, ***p<0.001.

### Viral protein p17 promotes autophagic engulfment of mitochondria by facilitating the interaction of SQSTM1 with TOMM70

**Figure 3.**
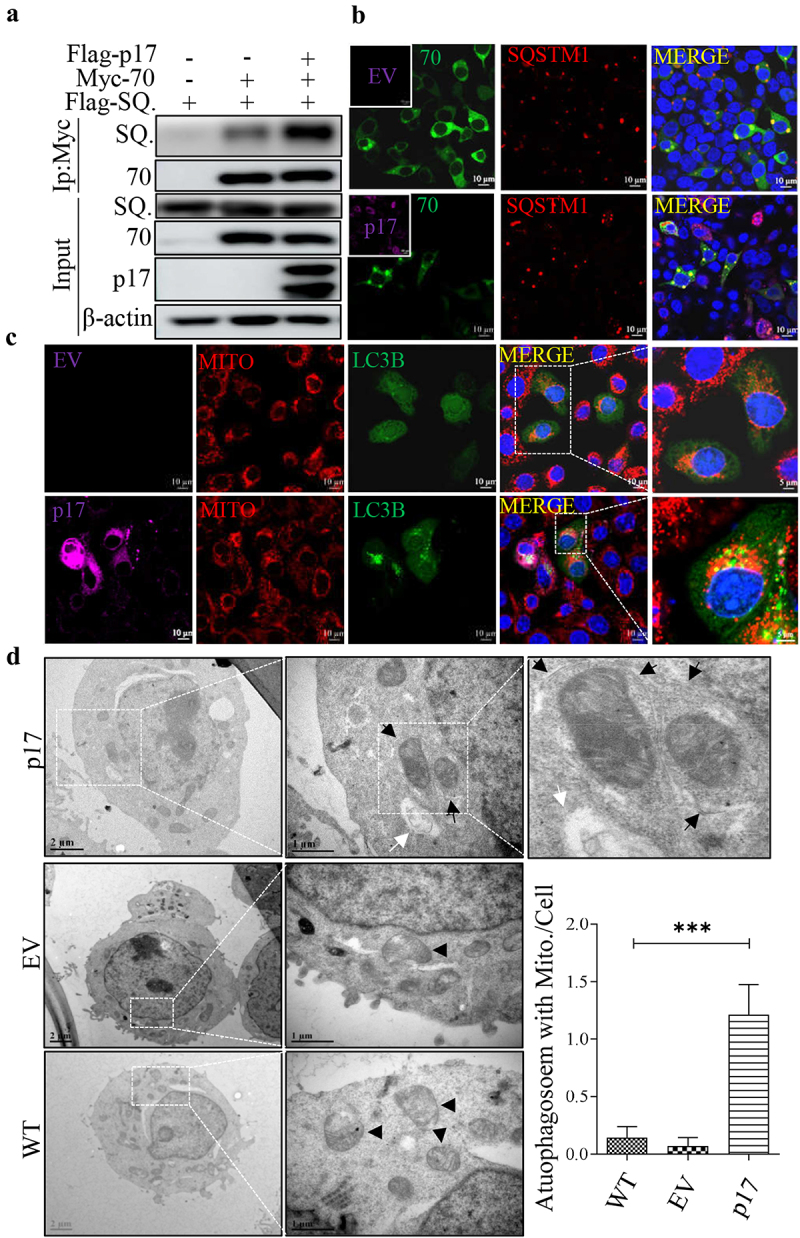
P17 promotes autophagic engulfment of mitochondria by facilitating the binding of SQSTM1 to TOMM70.

SQSTM1 is a classic autophagic receptor located throughout the cell and leads to the autophagic degradation of mitochondria [32]. We therefore tested whether p17 promotes the interaction between SQSTM1 and TOMM70. HEK-293T cells were cotransfected with vectors expressing Myc-TOMM70 and Flag-SQSTM1, with or without Flag-p17 expression. As shown in Figure 3a, p17 obviously enhanced the interaction between SQSTM1 and TOMM70. Next, we tested whether p17 promoted the colocalization of TOMM70 and SQSTM1 by confocal microscopy. As shown in Figure 3b, p17 enhanced the overlay between TOMM70 and SQSTM1. The above data suggested that p17 promoted the binding of SQSTM1 to TOMM70. Given that SQSTM1 is an autophagy cargo receptor, we wondered whether p17 regulates the inclusion of TOMM70 in the autophagosome. Confocal analysis suggested that colocalization between p17 and GFP-LC3B increased greatly in the presence of Flag-p17 (Figure 3c). According to transmission electron microscopy, p17 promoted the engulfment of mitochondria by autophagic vesicles, and the mitochondria are more damaged and swollen than the crest of the control group (Figures 3d), strongly suggesting that p17 promotes autophagic degradation of mitochondria.

(a) HEK-293T cells were cotransfected with Flag-SQSTM1 (Flag-SQ.) and Myc-TOMM70, together with empty vectors or Flag-p17. At 36 h post-transcription, cells were harvested for coimmunoprecipitation (Co-IP). WB assays were performed by using anti-Flag or anti-Myc antibodies. (b) HEK-293T cells were transfected with Flag-SQSTM1 and Myc-TOMM70, together with empty vectors or Flag-p17. At 24 h post-transcription, cells were harvested for confocal analysis. Scale bar: 5 µm. (c) 3D4/21 cells were cotransfected with GFP-LC3B and empty vector (EV) or Flag-p17. At 48 h post-transcription, cells were fixed and subjected to mitochondrial staining with MitoTracker Red CMXRos. Scale Bar: 10 µm. d HEK-293T cells were transfected with empty vectors or Flag-p17. At 36 h post-transcription, sample preparation was performed, and the samples were analysed with a transmission electron microscope. Double membrane vesicles are indicated with black arrows, and mitochondria are indicated with black arrowheads. The number of mitochondria with autophagosomes per cell was counted by counter, *n* > 15. At least three independent experiments were performed. The error bars show the standard error of the mean (SEM). Significance was analysed with two-tailed Student’s test. *p<0.05, **p<0.01, ***p<0.001.

### Viral protein p17 promotes mitochondrial degradation

**Figure 4.**
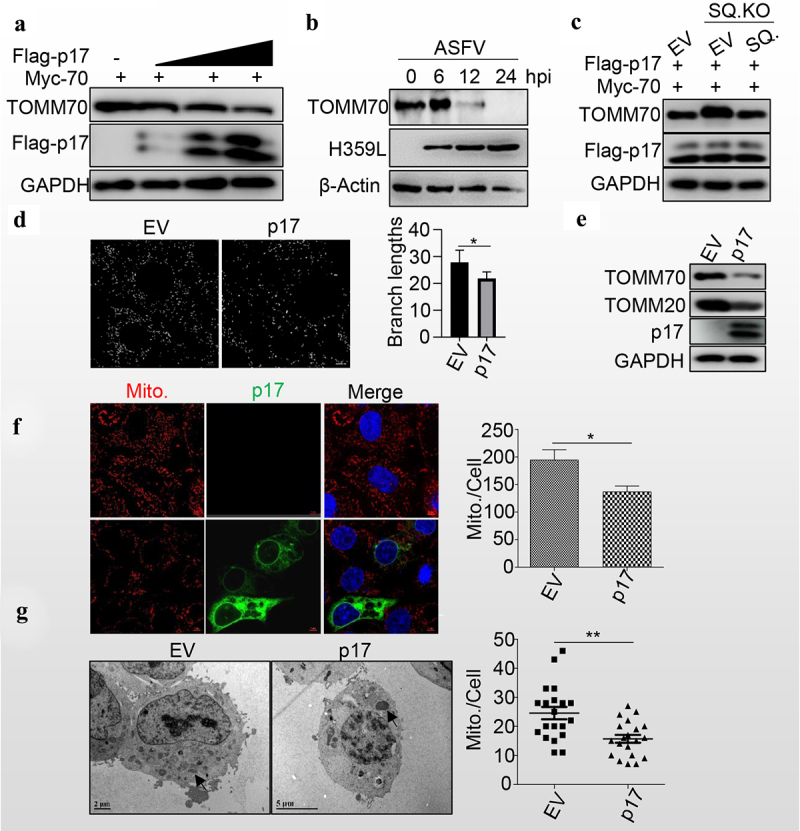
P17 promotes mitochondrial degradation.

Given that p17 promotes autophagy, we wondered whether p17 regulates the degradation of TOMM70. Interestingly, the levels of TOMM70 gradually decreased with the expression of p17 in a dose-dependent manner (Figure 4a). Similarly, the level of TOMM70 gradually decreased as the ASFV infection progressed (Figure 4b). Consistently, SQSTM1 knockout increased the level of TOMM70, while SQSTM1 expression in SQSTM1 KO cell lines rescued the degradation of TOMM70 (Figure 4c). Moreover, mitochondrial dynamics were analysed by using ImageJ-MiNA software, and the results showed that p17 expression significantly reduced the branch length, suggesting that p17 resulted in the fission of mitochondria (Figure 4d). Consequently, we observed that p17 expression decreased the levels of TOMM20 and TOMM70 (Figure 4e). Moreover, MitoTracker Red CMXRos staining showed that p17 expression significantly decreased the number of mitochondria (Figure 4f). Furthermore, the number of mitochondria was counted in empty vector- or p17-expressing cells by TEM analysis, and the data showed that p17 expression strongly decreased the number of mitochondria (Figure 4g), confirming that p17 promotes SQSTM1-mediated autophagic degradation of mitochondria.

(a) HEK-293T cells overexpressing TOMM70 were transfected with different amounts (0.5 μg, 1 μg, 2 μg) of empty vectors or Flag-p17. At 36 h post-transcription, cells were harvested, and cell lysates were then blotted with anti-TOMM70, anti-Flag, and anti-GAPDH antibodies. (b) PAM cells were infected with ASFV (MOI = 1) for indicated time and then harvested for WB analysis using anti-H359L polyclonal antibody, anti-TOMM70 and -β-Actin antibodies. (c) HEK-293T or SQSTM1 (SQ.) KO cells were transfected with Flag-p17 and Myc-TOMM70, either with empty vector or SQSTM1 (SQ.) as indicated for 36 h and were then harvested for WB analysis using anti-TOMM70 and anti-Flag antibodies. (d) 3D4/21 cells were transfected with Flag-p17 or empty vector for 48 hours and were then fixed with 4% paraformaldehyde, followed by mitochondrial staining with MitoTracker Red CMXRos and anti-Flag antibody. The images were captured by confocal microscopy. The mitochondrial branch length was analysed by using ImageJ-MiNA software. Significance was analysed with two-tailed Student’s test. *p < 0.05. Scale bar: 5 µm. (e) HEK-293T cells were transfected with empty vectors or Flag-p17. At 36 h post-transcription, cells were harvested, and cell lysates were then harvested for WB analysis using anti-TOMM70, anti-TOMM20, anti-Flag, and anti-GAPDH antibodies. (f) 3D4/21 cells were transfected with Flag-p17 or empty vector for 48 hours and were then fixed with 4% paraformaldehyde, followed by mitochondrial staining with MitoTracker Red CMXRos and anti-Flag antibody. The number of red dots/cells was counted by ImageJ and quantified. Scale bar: 5 µm. (g) HEK-293T cells were transfected with plasmids expressing ASFV p17 or empty vectors. At 36 h post-transcription, cells were harvested for TEM (transmission electron microscopy) analysis. Black arrows indicate mitochondria. The number of mitochondria/cell was counted by a counter, *n* > 15. At least three independent experiments were performed. The error bars show the standard error of the mean (SEM). Significance was analysed with two-tailed Student’s test. *p<0.05, **p<0.01, ***p<0.001.

### Viral protein p17 inhibits the innate immune response by degrading MAVS

**Figure 5.**
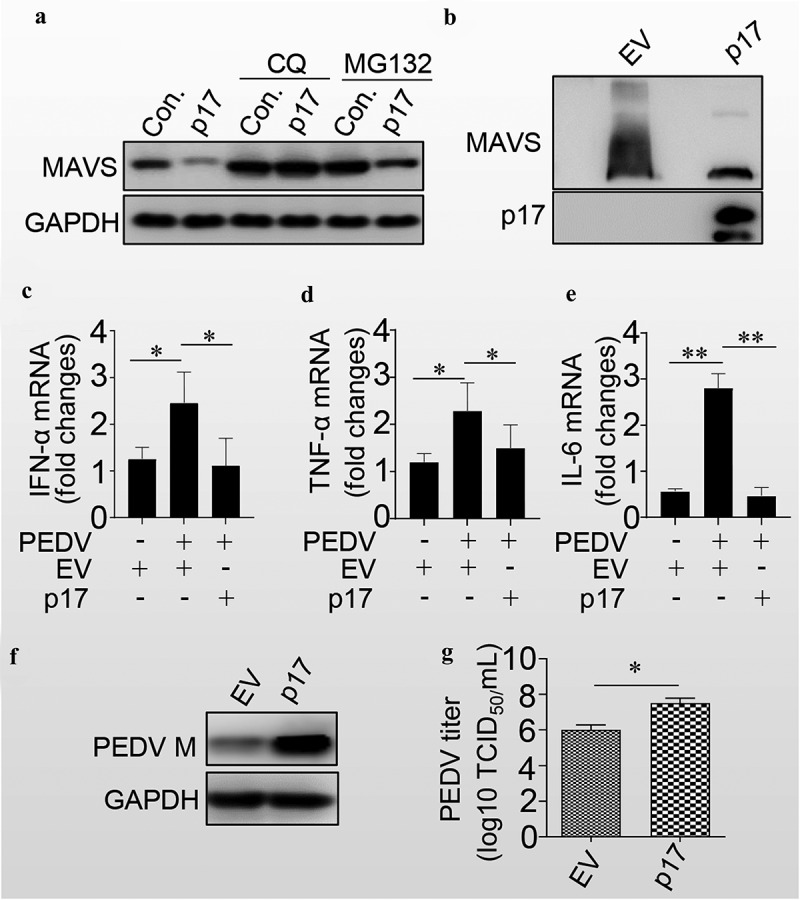
P17 inhibits the innate immune response by autophagic degradation of MAVS.

Since p17 induced the degradation of mitochondria and mitochondrial antiviral-signalling protein (MAVS) localized at the outer membrane of mitochondria, we next tested whether p17 promoted the degradation of MAVS. As shown in Figure 5a, p17 expression enhanced MAVS degradation. However, treatment with CQ, but not MG132, blocked the p17-mediated degradation of MAVS, suggesting that p17 induced the degradation of MAVS through autophagy. We also found that p17 expression significantly reduced the aggregation of MAVS (Figure 5b). We next tested the effect of p17 on the level of IFN-α. As shown in Figure 5c-e, PEDV indeed significantly increased the levels of IFN-α, TNF-α and IL-6, while p17 expression counteracted the effect of PEDV-mediated induction of these innate immunity factors. Consequently, as expected, p17 indeed increased the level of PEDV M (Figure 5f), as well as the virus titres (Figure 5g). The above data strongly suggest that p17 is beneficial for RNA virus replication by promoting the degradation of MAVS, MDA5 and RIGI.

(a) HEK-293T cells were transfected with empty vector or vectors expressing MAVS, MDA5, and RIGI in the presence or absence of CQ or MG132. The cells were harvested for WB analysis by using anti-MAVS, MDA5, and RIGI antibodies. (b) HEK-293T cells were transfected with empty vector or vectors expressing Flag-p17. The cells were harvested for WB analysis by using anti-MAVS or anti-Flag antibodies. (c, d and e) VERO cells were transfected with empty vector or vector expressing p17 for 24 hours and were then infected with PEDV for 24 hours. The cells were then harvested for qPCR analysis. (f) Vero cells were transfected with empty vector or vector expressing p17 and were then harvested for WB analysis by using anti-PEDV M and GAPDH monoclonal antibodies. (g) Vero cells transfected with EV or vector expressing p17 were infected with PEDV, and the TCID_50_ was then tested. At least three independent experiments were performed. The error bars show the standard error of the mean (SEM). At least three independent experiments were performed. The error bars show the standard error of the mean (SEM). Significance was analysed with two-tailed Student’s test. *p<0.05, **p<0.01, ***p<0.001.

## Discussion

ASFV causes severe leucopenia and leads to massive destruction of lymphoid organs and tissues. In general, the above pathogenesis is believed to result from cell death [33–36]. However, similar to other viruses, ASFV exploits multiple strategies to inhibit apoptotic pathways to allow complete virus replication. Several ASFV-encoding viral proteins, such as pA179L, pA224L, pEP153R and pDP71L, play critical roles in inhibiting apoptosis [37–41]. p17 has no effect on inducing apoptosis. In our study, we showed that p17 promoted the degradation of TOMM70, which has been reported to promote SeV-induced apoptosis [42], suggesting that the removal of TOMM70 and damaged mitochondria are likely to prevent apoptosis during ASFV infection.

TOMM70 not only acts as an input receptor for the outer membrane protein of the mitochondria, which relies on maintaining the basic morphological structure of the mitochondria, but also plays a variety of roles in innate immunity and maintenance of cellular homoeostasis [43]. The interaction of TOMM70 and Hsp90 activates IRF3 and IRF-mediated antiviral responses [44]. TOMM70 functions as a receptor of the mitochondrial antiviral-signalling protein (MAVS) and thereby participates in the corresponding innate immune response against viral infections [45]. Additionally, the TOMM core complex, which includes TOMM70, 40 and 22, is critical for Parkin-induced mitophagy [15,16]. In the present study, we found that ASFV p17 promotes mitophagy by facilitating the binding of SQSTM1 to TOMM70, which is a novel role for p17 as an adaptor for mitophagy. Hence, we might expect that TOMM subunits act as adaptors for mitophagy.

The mitochondrial outer membrane provides MAVS, an essential component of the innate immune pathway, mediates innate immunity against RNA viruses. Several viruses have consequently developed their own strategies to regulate mitophagy to dampen host antiviral immunity. HPIV3, measles virus (MeV), IAV, EBV, and HTNV block the host type I IFN response by inducing mitophagy and facilitating MAVS degradation [46–50]. In our study, p17-induced mitophagy facilitated the inhibition of the type I IFN response induced by PEDV infection, implying the possibility that viruses benefit each other during infection.

Autophagy is used by cells to break down viral particles. ASFV downregulates the autophagy-related genes Atg2A, Atg9A, Atg101, Atg4B and Atg7; upregulates nuclear protein 1 (NUPR1), which inhibits autophagy; affects bnip3 expression in infected cells [51] activates the mTORC1-AKT pathway, which negatively regulates autophagy [52] and inhibits autophagy during the early stage of ASFV infection [53], suggesting that autophagy probably plays an antiviral role during ASFV infection. However, evidence that autophagy inhibits ASFV infection is still lacking, and whether p17-induced autophagy mediates the antiviral response is also unknown. Therefore, further study on the interaction between autophagy and ASFV is necessary to deepen our understanding of the immune response to ASFV infection.

## Materials and methods

### Cell cultures

HEK-293T cells, Vero cells and 3D4/21 cells were cultured in Dulbecco’s modified Eagle medium (DMEM; Gibco, Carlsbad, CA USA) supplemented with 10% foetal bovine serum (FBS; 1616756, Biological Industries, Israel).

### Western blotting

Cells were harvested at indicated time points and lysed immediately in lysis buffer containing 2% sodium dodecyl sulphate [SDS], 50 mM Tris-HCl, 1% Triton X-100, 150 mM NaCl with pH 7.5. The lysate proteins were separated though SDS-polyacrylamide gel electrophoresis (PAGE), and the proteins were then transferred onto nitrocellulose blotting membranes (NC) (10600001; GE Healthcare Life Science). The membranes were blocked with 5% non-fat dry milk and 0.1% Tween 20 for 30 min at 37 °C, and then incubated with primary antibodies for 2 h at 37 °C, followed by incubating with horseradish peroxidase-conjugated anti-mouse/rabbit IgG (Kirkegaard & Perry Laboratories, Inc., 074–1506), finally imaged using AI680 Images (GE Healthcare, USA) after using enhanced chemiluminescence (ECL).

### Cell transfection and treatment

For cell transfection, HEK-293T or 3D4/21 cells were seeded on the designated plates or glass coverslips according to the experimental scheme. When cells were 70–80% confluent, they were transfected with ExFect™ Transfection Reagent. After transfection with vectors for 24 h, the cells were harvested for immunoblotting to measure the protein levels.

### Immunofluorescence staining and confocal microscopy

The indicated HEK-293T or 3D4/21 cells were seeded on 35 mm glass-bottomed cell culture dishes for 24 h, and then transfected with the indicated plasmids, in the presence or absence of the indicated drugs, or cultured under starvation conditions for indicated time. After fixing using 4% paraformaldehyde for 10 min at room temperature, the cells were then blocked and permeabilized with 5% non-fat milk with 0.2% Triton X-100 for 1 h. The cell samples were then incubated with the indicated antibodies for 2 h at 37 °C after washing three times with PBST, and with another three times of washing with PBST, the samples were then incubated with DyLight 405-, FITC-, Alexa Fluor 647-, or Alexa Fluor 546-labelled IgG antibodies for 1 h. After washing three times with PBST and stained with DAPI indicating nuclei, and the cells were observed under an N-structured illumination microscopy (SIM) Super-Resolution microscope (Nikon, Tokyo, JPN) or a Nikon A1R/A1 laser scanning confocal microscope (Nikon, Tokyo, JPN). Only cells with more than GFP dots or ring-like structures were scored as positive.

### Coimmunoprecipitation assay

Cell lines were separately transfected with various vectors together or singly for indicated time and then lysed using NP40 lysis buffer with phenylmethanesulfonyl fluoride (PMSF, 1 mM) for 4 h at 4 °C. Then, the supernatants from the cell lysis after centrifuging at 12,000 × g for 10 min, followed by incubation with anti-Flag antibody and Protein A/G beads for 4 h at 4 °C. The supernatants were removed after centrifugation, and the pellets were suspended in washing buffer. This step were repeated for 5 times. The pellets were finally lysed in lysis buffer for WB analysis.

### RNA extraction and quantitative real-time PCR

Total RNA was extracted following the instructions of the TRIzol reagent, and the cDNA were generated after reverse transcription of RNA a reverse transcription kit. Quantitative real-time PCR was performed using AceQ qPCR SYBR Green Master Mix with specific primers and a Roche LC96 real-time PCR system.

### TEM sample preparation

The cells were fixed with 2.5% glutaraldehyde at 4 °C overnight. The cell pellets were then acquired for agarose embedding. After the agarose cooled, the cell samples were cut into small pieces and then rinsed using phosphate buffer (0.1 M, pH 7.0) 3 times. The samples were fixed using 1% osmium acid solution for 1.5 h. Then, the osmium acid solution was discarded, and the samples were washed with phosphoric acid buffer 3 times for 15 min each. The samples were then dehydrated with different concentrations of ethanol solutions, namely, 30%, 50%, 70%, 80%, for 15 min each, and then, 90% and 95% acetone solutions were added and incubated with the samples to dehydrate them for 15 min each. Finally, the samples were treated twice with pure acetone solution for 20 min each. The samples were treated with Spur embedding agent and acetone at a ratio of 1:1 at room temperature for 1 h, the samples were treated with Spur embedding agent and acetone at a ratio of 3:1 at room temperature for 3 h, and finally, the samples were treated with pure embedding agent overnight at room temperature. Finally, the samples were labelled for observation.

Then, these samples were observed using a Hitachi H-9500 transmission electron microscope (Hitachi High-Technologies Corporation) at 80 kV.

### Statistical analysis

The western blotting and Co-IP results are representative of three independent experiments. All the statistical analyses were performed using Prism 8 (Graph Pad). Student’s t test was used to evaluate the statistical significance, and *p* < 0.05 was considered statistically significant.

## Data Availability

The authors confirm that the data supporting the findings of this study are available within the article.
